# Mistreatment Experiences, Protective Workplace Systems, and Occupational Distress in Physicians

**DOI:** 10.1001/jamanetworkopen.2022.10768

**Published:** 2022-05-06

**Authors:** Susannah G. Rowe, Miriam T. Stewart, Sam Van Horne, Cassandra Pierre, Hanhan Wang, Makaila Manukyan, Megan Bair-Merritt, Aviva Lee-Parritz, Mary P. Rowe, Tait Shanafelt, Mickey Trockel

**Affiliations:** 1 Office of Equity, Vitality and Inclusion, Boston University Medical Group, Boston Medical Center, Boston, Massachusetts; 2 Boston University School of Medicine, Boston, Massachusetts; 3 Children's Hospital of Philadelphia, Perelman School of Medicine at the University of Pennsylvania, Philadelphia; 4 Center for WorkLife Wellbeing, ChristianaCare, Wilmington, Delaware; 5 Stanford University School of Medicine, Stanford, California; 6 Sloan School of Management, Massachusetts Institute of Technology, Cambridge

## Abstract

**Importance:**

Reducing physician occupational distress requires understanding workplace mistreatment, its relationship to occupational well-being, and how mistreatment differentially impacts physicians of diverse identities.

**Objectives:**

To assess the prevalence and sources of mistreatment among physicians and associations between mistreatment, occupational well-being, and physicians’ perceptions of protective workplace systems.

**Design, Setting, and Participants:**

This survey study was administered in September and October 2020 to physicians at a large academic medical center. Statistical analysis was performed from May 2021 to February 2022.

**Main Outcomes and Measures:**

Primary measures were the Professional Fulfillment Index, a measure of intent to leave, and the Mistreatment, Protection, and Respect Measure (MPR). Main outcomes were the prevalence and sources of mistreatment. Secondary outcomes were the associations of mistreatment and perceptions of protective workplace systems with occupational well-being.

**Results:**

Of 1909 medical staff invited, 1505 (78.8%) completed the survey. Among respondents, 735 (48.8%) were women, 627 (47.1%) were men, and 143 (9.5%) did not share gender identity or chose “other”; 12 (0.8%) identified as African American or Black, 392 (26%) as Asian, 10 (0.7%) as multiracial, 736 (48.9%) as White, 63 (4.2%) as other, and 292 (19.4%) did not share race or ethnicity. Of the 1397 respondents who answered mistreatment questions, 327 (23.4%) reported experiencing mistreatment in the last 12 months. Patients and visitors were the most common source of mistreatment, reported by 232 physicians (16.6%). Women were more than twice as likely as men to experience mistreatment (31% [224 women] vs 15% [92 men]). On a scale of 0 to 10, mistreatment was associated with a 1.13 point increase in burnout (95% CI, 0.89 to 1.36), a 0.99-point decrease in professional fulfillment (95% CI, −1.24 to −0.73), and 129% higher odds of moderate or greater intent to leave (odds ratio, 2.29; 95% CI, 1.75 to 2.99). When compared with a perception that protective workplace systems are in place “to a very great extent,” a perception that there are no protective workplace systems was associated with a 2.41-point increase in burnout (95% CI, 1.80 to 3.02), a 2.81-point lower professional fulfillment score (95% CI, −3.44 to −2.18), and 711% higher odds of intending to leave (odds ratio, 8.11; 95% CI, 3.67 to 18.35).

**Conclusions and Relevance:**

This survey study found that mistreatment was common among physicians, varied by gender, and was associated with occupational distress. Patients and visitors were the most frequent source, and perceptions of protective workplace systems were associated with better occupational well-being. These findings suggest that health care organizations should prioritize reducing workplace mistreatment.

## Key Points

**Question:**

What are the prevalence and common sources of workplace mistreatment of physicians, and is there an association between workplace mistreatment and occupational well-being?

**Findings:**

A survey of 1505 physicians conducted from September to October 2020 found that 23.4% had experienced mistreatment in the last year, with patients and visitors as the most frequent source of mistreatment. Mistreatment was associated with higher levels of occupational distress, whereas the perception that protective workplace systems exist was associated with lower levels of occupational distress.

**Meaning:**

These findings suggest that systems that prevent workplace mistreatment may improve physicians’ occupational well-being.

## Teaser

This survey study evaluates the prevalence and sources of mistreatment among physicians and associations between mistreatment, occupational well-being, and physicians’ perceptions of protective workplace systems.

## Introduction

Studies of mistreatment in medicine have largely focused on mistreatment among trainees, with less attention on practicing physicians.^1,2,3,4,5,6,7^ Among trainees, mistreatment is common and varies by gender and race; in a national survey of general surgery residents, almost two-thirds of women reported experiencing gender-based harassment, compared with 10% of men.^4^ Mistreatment of physicians of color may be more common, although the literature is sparse. Limited data suggest that workplace mistreatment experiences are associated with increased burnout,^4^ worse job performance,^8^ and depression.^1^

Sources of mistreatment in health care include colleagues, patients and visitors, and for trainees, supervising physicians.^4,6,9,10^ However, few studies have directly addressed mistreatment by patients and visitors.

We explored the prevalence and sources of mistreatment among physicians at a large academic medical center and assessed how mistreatment experiences vary by gender and race. We also studied the association of experiencing mistreatment with burnout, professional fulfillment, and intent to leave the organization. Finally, we evaluated the hypothesis that physicians’ perceptions of protective workplace systems are associated with occupational well-being.

## Methods

### Survey Administration

In September and October of 2020, Stanford University School of Medicine, Stanford, California, conducted an administrative survey through an independent third-party survey administrator to inform organizational efforts to improve professional fulfillment and wellness among clinical faculty. All Stanford-employed members of the clinically active medical staff with an appointment of at least 0.5 full-time equivalent were invited to participate via email. Up to 5 reminders were sent to nonrespondents. The 3 departments with the highest response rates received $50 per respondent. All respondents had MD or DO degrees, with the exception of 20 clinical psychologists. To simplify, we refer to the medical staff as physicians from this point on.

Participation was voluntary. The response rate of complete and partially completed surveys was determined using the American Association for Public Opinion Research (AAPOR) reporting guideline.^11,12^ The Stanford University institutional review board deemed this study exempt and informed consent was not needed because the study involved retrospective analysis of administratively collected deidentified data.

### Survey Measures

#### Occupational Well-being

The survey included the Professional Fulfillment Index. This index is a measurement of burnout and professional fulfillment demonstrated to have good reliability and construct validity, along with a standardized question about intent to leave within the next 2 years, described elsewhere.^13,14,15^

#### Measures of Mistreatment and Protections From Mistreatment

The Mistreatment, Protection, and Respect (MPR) Measure is a 7-item measure assessing experiences of different types of mistreatment, together with the sources of mistreatment, as well as the perception of protective workplace systems. The MPR was created by the authors by conducting literature searches for relevant theoretical frameworks and related measures. The survey was then reviewed for face and content validity by 7 researchers with extensive experience in Diversity Equity and Inclusion (DEI) in the health care setting. After iterative feedback and improvement, the scale was pilot tested with a diverse sample of 16 clinicians working in the area of DEI (additional details in eAppendix 1 of the Supplement). Respondents answered yes or no to the question: “Have you experienced the following at work in the last 12 months and if so from whom?” for 3 categories of mistreatment: sexual harassment or abuse; verbal mistreatment or abuse; physical intimidation, violence, or abuse. Possible sources included: patient/family/visitors; colleague; nurse; other staff; and/or leadership. Participants could choose all that apply.

The MPR also includes 2 questions related to protective factors, measured on a 5-point Likert scale (not at all = 0; to a very great extent = 4): “There are good systems in place to ensure that I am treated with respect and dignity” and “Bystanders speak up or intervene if someone is mistreated.” The MPR also includes 2 questions not addressed in this study. (See the eAppendix 1 in the Supplement for a description of the methodology used to develop the MPR measure, and see eAppendix 2 in the Supplement for the full text of the measure.)

#### Demographics

Demographic characteristics were assessed including self-reported gender, race, age, and specialty. Respondents were asked to choose one gender from options: female, male, self-defined, prefer not to answer. Respondents selected one or more race categories from these options: Asian, Black, White, Other, prefer not to answer.

### Statistical Analysis

Descriptive statistics were reported to summarize respondent characteristics. Percentages were reported for categorical variables. Means and standard deviations were reported for continuous variables. Professional fulfillment and burnout were scored using a published approach and transformed to a 10-point scale.^16^ Differences in responses to the mistreatment questions among gender and racial groups were examined using a Pearson χ^2^ test. Associations between mistreatment and burnout and mistreatment and professional fulfillment were examined using linear regression models. The difference in burnout and professional fulfillment by mistreatment experience was further examined by multiple linear regression models where we added responses to the questions assessing perceptions that “bystanders speak up or intervene” and “there are good systems in place to ensure that I am treated with respect and dignity.” To assess standardized mean difference (SMD) effect size associations with independent variables, multiple linear regression models were repeated with standardized (*z* score) transformed dependent variables for both burnout and professional fulfillment. To examine the association between intent to leave and mistreatment experience, we used univariable logistic regression, and then used multiple logistic regression with responses to questions assessing perceptions that “bystanders speak up or intervene” and “there are good systems in place to ensure that I am treated with respect and dignity.” All analyses were conducted in R version 3.6.0 (R Project for Statistical Computing) from May 2021 to February 2022. A 2-sided *P* ≤ .05 was considered statistically significant.

## Results

Of 1909 physicians invited, 1505 (78.8%) responded, including 735 women (48.8%), 627 men (41.7%), and 143 (9.5%) whose gender either was not disclosed or who identified as neither male nor female (<5 respondents); 12 (0.8%) identified as African American or Black, 392 (26%) as Asian, 10 (0.7%) as multiracial, 736 (48.9%) as White, 63 (4.2%) as other, and 292 (19.4%) did not share race or ethnicity (Table 1).

**Table 1.** Descriptive Statistics for Respondents

Characteristic	Respondents, No. (%) (N = 1505)
Department	
Anesthesiology	160 (10.6)
Cardiothoracic surgery	14 (0.9)
Dermatology	37 (2.5)
Emergency medicine	71 (4.7)
Medicine	305 (20.3)
Neurology	67 (4.5)
Neurosurgery	16 (1.1)
OBGYN	36 (2.4)
Ophthalmology	28 (1.9)
Orthopaedic surgery	58 (3.9)
Otolaryngology	36 (2.4)
Pathology	56 (3.7)
Pediatrics	301 (20.0)
Psychiatry	102 (6.8)
Radiation oncology	37 (2.5)
Radiology	87 (5.8)
Prefer not to say	3 (0.2)
Surgery	74 (4.9)
Urology	17 (1.1)
Burnout, score on 0-10 scale, mean (SD)^a^	3.04 (1.96)
Professional fulfillment, score on 0-10 scale, mean (SD)^b^	6.53 (2.09)
Intent to leave	
No	1047 (69.6)
Yes	366 (24.3)
Missing	92 (6.1)
Gender	
Female	735 (48.8)
Male	627 (41.7)
Missing^c^	143 (9.5)
Race	
African American or Black	12 (0.8)
Asian	392 (26.0)
Multiracial	10 (0.7)
White	736 (48.9)
Other^d^	63 (4.2)
Missing	292 (19.4)
Protective systems	
Not at all	62 (4.1)
To a small extent	153 (10.2)
To a moderate extent	383 (25.4)
To a great extent	508 (33.8)
To a very great extent	278 (18.5)
Missing	121 (8.0)
Bystanders speak up	
Not at all	100 (6.6)
To a small extent	269 (17.9)
To a moderate extent	453 (30.1)
To a great extent	369 (24.5)
To a very great extent	160 (10.6)
Missing	154 (10.2)
Experienced mistreatment^e^	
No	1070 (71.1)
Yes	327 (21.7)
Missing	108 (7.2)

^a^ Higher scores unfavorable.

^b^ Higher scores favorable.

^c^ Missing gender includes respondents who elected not to identify their gender and less than 5 respondents who self-identified as a less-represented gender.

^d^ Respondents were given the option to select “other” in describing their race.

^e^ Includes any respondent who reported experiencing at least 1 form of mistreatment (sexual, verbal, or physical).

Of the physicians who responded to questions on mistreatment, 327 of 1397 (23.4%) reported experiencing workplace mistreatment in the past 12 months (Table 2). Mistreatment by patients and visitors was reported by 232 physicians (16.6%), representing the most common source of mistreatment at 70.9% of all mistreatment events. Other physicians were the second most common source of mistreatment, reported by 7.1% of respondents. Verbal mistreatment was the most frequent form of mistreatment, reported by 298 respondents (21.5%), followed by sexual harassment (74 respondents [5.4%]), and physical intimidation or abuse (72 respondents [5.2%]).

**Table 2.** Type and Source of Mistreatment^a^

Type of mistreatment	No.	No. (%)					
		Any source	Patient/family/visitors	Colleague	Nurse	Other staff	Leadership
Sexual harassment or abuse	1384	75 (5.4)	56 (4.0)	21 (1.5)	1 (0.1)	8 (0.6)	6 (0.4)
Verbal mistreatment or abuse	1386	298 (21.5)	203 (14.6)	83 (6.0)	15 (1.1)	21 (1.5)	41 (3.0)
Physical intimidation, violence, or abuse	1394	72 (5.2)	65 (4.7)	5 (0.4)	2 (0.1)	3 (0.2)	1 (0.1)
Any of above forms of mistreatment^b^	1397	327 (23.4)	232 (16.6)	99 (7.1)	17 (1.2)	29 (2.1)	43 (3.1)

^a^ Percentages in each column or row may add up to more than 100%, as individual respondents may have endorsed mistreatment in multiple categories and/or from multiple sources.

^b^ Includes any respondent who reported experiencing at least 1 form of mistreatment (sexual, verbal, or physical).

Table 3 reports the prevalence of mistreatment experiences by gender and race. Mistreatment experiences differed significantly by gender, with a greater proportion of women (63 of 715 [8.8%]) than men (9 of 609 [1.5%]) reporting experiencing sexual harassment (*P* < .001; χ^2^_1_ = 32.98). Women were also more likely (201 of 717 [28.0%]) than men (87 of 609 [14.3%]) to report experiencing verbal mistreatment (*P* < .001; χ^2^_1_ = 35.80). Overall, 224 women (31.0%) experienced 1 or more forms of mistreatment compared with 92 men (15.0%) (*P* < .001; χ^2^_1_ = 46.61).

**Table 3.** Experience of Mistreatment by Gender and Race

Type of mistreatment	Gender			Race					
	No. (%)		*P* value	No. (%)					*P* value
	Female	Male		Black	Asian	Multiracial	White	Other	
Sexual harassment or abuse									
No	652 (91.2)	600 (98.5)	<.001	11 (91.7)	366 (95.8)	9 (90.0)	683 (95.1)	54 (87.1)	.06
Yes	63 (8.8)	9 (1.5)		1 (8.3)	16 (4.2)	1 (10.0)	35 (4.9)	8 (12.9)	
Verbal mistreatment or abuse									
No	516 (72.0)	522 (85.7)	<.001	6 (50.0)	297 (77.7)	5 (55.6)	587 (81.4)	42 (68.9)	.003
Yes	201 (28.0)	87 (14.3)		6 (50.0)	85 (22.3)	4 (44.4)	134 (18.6)	19 (31.1)	
Physical intimidation, violence, or abuse									
No	675 (93.9)	590 (95.9)	.12	10 (83.3)	366 (95.3)	8 (80.0)	689 (95.3)	55 (88.7)	.01
Yes	44 (6.1)	25 (4.1)		2 (16.7)	18 (4.7)	2 (20.0)	34 (4.7)	7 (11.3)	
Any of above forms of mistreatment									
No	498 (69.0)	523 (85.0)	<.001	6 (50.0)	295 (76.4)	5 (50.0)	572 (79.0)	42 (67.7)	.008
Yes	224 (31.0)	92 (15.0)		6 (50.0)	91 (23.6)	5 (50.0)	152 (21.0)	20 (32.3)	

Statistically significant disparities in workplace mistreatment were present across racial groups (*P* = .008; χ^2^_4_ = 13.79) (Table 3). Differences by race were demonstrated in (1) verbal mistreatment (*P* = .003; χ^2^_4_ = 15.78), and (2) physical intimidation or violence (*P* = .01; χ^2^_4_ = 12.65). These analyses comparing distribution of mistreatment across racial groups do not attempt to contrast specific pairs of racial categories for statistically significant differences. However, descriptive mistreatment data for racial subgroups is summarized below.

Multiracial and Black physicians were more likely than White and Asian physicians to report experiencing at least 1 form of mistreatment. Experiencing verbal mistreatment was highest among Black physicians (6 of 12 [50%]) and lowest among White physicians (134 of 721 [18.6%]). Being subjected to physical intimidation or abuse was highest among multiracial physicians (2 of 10 [20%]) and lowest among White (34 of 723 [4.7%]) and Asian (18 of 384 [4.7%]) physicians. Experiencing any type of mistreatment was most common among multiracial physicians (5 of 10 [50.0%]) and Black physicians (6 of 12 [50.0%], and least common among White physicians (152 of 724 [21.0%]). A subset analysis comparing people who did not provide gender or race and ethnicity data with those who did yielded no significant differences in reports of mistreatment (eTable1 in the Supplement).

### Results of Regression Analyses

Regression analyses (Table 4) found that having experienced any type of workplace mistreatment was associated with a 1.13-point increase in burnout (scale range: 0 to 10; 95% CI, 0.89 to 1.36; *P* < .001) and a 0.99-point (scale range: 0 to 10) decrease in professional fulfillment (95% CI, −1.24 to −0.73; *P* < .001). Expressed in terms of standardized mean difference, having experienced any type of workplace mistreatment was associated with a 0.57 standard deviation unit increase in burnout score (95% CI, 0.45 to 0.70) and a 0.47 standard deviation unit decrease in the professional fulfillment (95% CI, −0.59 to −0.35) (eTable 2 in the Supplement). The association between burnout and mistreatment remained statistically significant after adjusting for the perception that bystanders intervene and that protective workplace systems are in place; however, the association with professional fulfillment of workplace mistreatment was no longer significant after adjusting for the perception that bystanders intervene and the perception that protective workplace systems are in place.

**Table 4.** Parameter Estimates From Regression Analyses of Associations of Mistreatment and Protective Factors With Burnout, Professional Fulfillment, and Intent to Leave

Independent variables	Linear regression, β (95% CI)		Model 3a (Intent to Leave) logistic regression, OR (95% CI)^c^
	Model 1a (burnout)^a^	Model 2a (PF)^b^	
Mistreatment			
No	[Reference]	[Reference]	1 [Reference]
Yes	1.13 (0.89 to 1.36)	−0.99 (−1.24 to −0.73)	2.29 (1.75 to 2.99)
Missing	0.36 (−0.07 to 0.79)	−0.12 (−0.56 to 0.33)	1.73 (0.83 to 3.43)
Abuse			
No	[Reference]	[Reference]	1 [Reference]
Yes	0.52 (0.29 to 0.76)	−0.20 (−0.45 to 0.04)	1.45 (1.08 to 1.94)
Missing	0.24 (−0.32 to 0.81)	−0.30 (−0.28 to 0.88)	1.73 (0.75 to 3.83)
Protective systems			
To a very great extent	[Reference]	[Reference]	1 [Reference]
To a great extent	0.76 (0.44 to 1.08)	−0.88 (−1.21 to −0.55)	1.93 (1.16 to 3.3)
To a moderate extent	1.30 (0.94 to 1.67)	−1.60 (−1.98 to −1.22)	2.76 (1.58 to 4.95)
To a small extent	1.71 (1.24 to 2.17)	−2.33 (−2.82 to −1.85)	4.65 (2.43 to 9.11)
Not at all	2.41 (1.80 to 3.02)	−2.81 (−3.44 to −2.18)	8.11 (3.67 to 18.35)
Missing	0.71 (0.04 to 1.38)	−0.98 (−1.67 to −0.28)	2.98 (1.14 to 7.63)
Bystanders speak up			
To a very great extent	[Reference]	[Reference]	1 [Reference]
To a great extent	0.30 (−0.09 to 0.69)	−0.34 (−0.74 to 0.07)	0.86 (0.47 to 1.59)
To a moderate extent	0.32 (−0.09 to 0.73)	−0.37 (−0.80 to 0.06)	0.78 (0.41 to 1.48)
To a small extent	0.27 (−0.19 to 0.74)	−0.47 (−0.95 to 0.01)	0.9 (0.45 to 1.78)
Not at all	1.08 (0.50 to 1.65)	−1.25 (−1.85 to −0.65)	1.53 (0.7 to 3.38)
Missing	0.58 (−0.03 to 1.19)	−0.92 (−1.55 to −0.29)	0.62 (0.25 to 1.5)

Abbreviations: OR, odds ratio; PF, professional fulfillment.

^a^ Data are for 1458 participants; *R*^2^ = 0.06; *F*_2,1455_ = 43.42.

^b^ Data are for 1479 participants; *R*^2^ = 0.04; *F*_2,1476_ = 29.18.

^c^ Data are for 1413 participants; Akaike information criterion = 1586.2.

In multivariable models (Table 4), decreased perception that protective workplace systems are in place was associated with higher levels of burnout and lower levels of professional fulfillment. Compared with the highest rating of protective workplace systems (systems in place “to a very great extent”), the lowest rating (systems in place “not at all”) was associated with a 2.41-point increase in burnout (95% CI, 1.80 to 3.02; *P* < .001) and a 2.81-point decrease in professional fulfillment (95% CI, −3.44 to −2.18; *P* < .001). Compared with the highest rating of bystander intervention (bystanders intervene “to a very great extent”), the lowest rating (bystanders intervene “not at all”) was associated with a 1.08-point increase in burnout (95% CI, 0.50 to 1.65; *P* = .002) and a 1.25-point decrease in professional fulfillment (95% CI, −1.85 to −0.65; *P* < .001). Smaller differences in perception that bystanders intervene did not demonstrate significant association with either burnout or professional fulfillment.

Any form of mistreatment was associated with 129% higher odds of reporting moderate or greater intent to leave within 2 years (odds ratio, 2.29; 95% CI, 1.75 to 2.99; *P* < .001) (Table 4). The association of mistreatment with intent to leave remained statistically significant after adjusting for the perception that bystanders intervene and that protective workplace systems are in place. In the multivariable model, decreased perception that protective workplace systems are in place was associated with greater intent to leave. Compared with the highest rating of protective workplace systems (systems in place “to a very great extent”), the lowest rating (systems in place “not at all”) was associated with 711% higher odds of moderate or greater intent to leave (odds ratio, 8.11; 95% CI, 3.67 to 18.35; *P* < .001). Differences in perception that bystanders intervene did not demonstrate significant association with intent to leave.

## Discussion

This survey study found a high prevalence of mistreatment among attending physicians, particularly women. Our study builds on existing literature on physician mistreatment in several ways. Although it has been reported that medical students and residents experience frequent mistreatment,^1,2,3,4,5,6,7,8,10^ to date there has been sparse corresponding data on the prevalence of sources of mistreatment for practicing physicians.^17,18,19,20^ We found that mistreatment was most likely to originate from patients and visitors, underscoring the need to address this less studied source of mistreatment. Finally, we found a strong association between mistreatment and worse occupational well-being, including increased burnout, reduced professional fulfillment, and higher reported intent to leave the organization. Conversely, having systems in place that protect physicians from mistreatment is associated with increased occupational well-being, both for those who experienced mistreatment and those who did not (Figure). To our knowledge, this is the first study to explore the association between the perception of protective workplace systems and occupational well-being for physicians.

**Figure.**
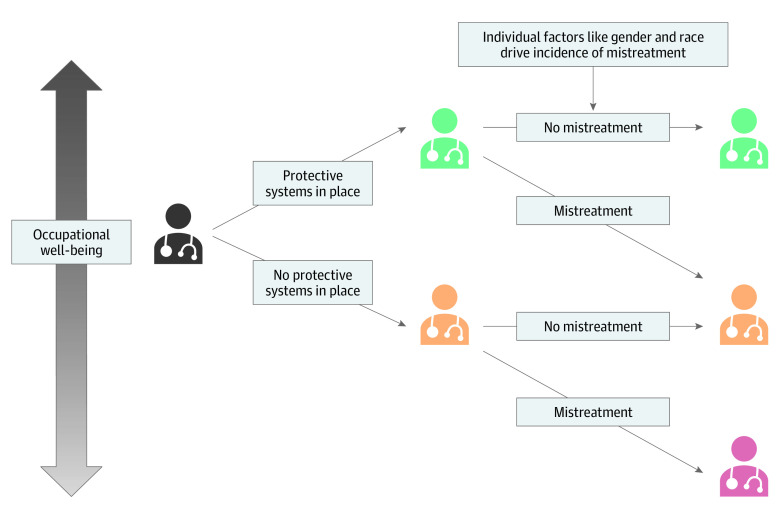
Conceptual Model of Association of Protective Workplace System and Mistreatment With Physician Occupational Well-being

Our finding that patients and visitors were the most frequent perpetrators of mistreatment toward physicians has important implications for physician well-being. Organizational interventions that address mistreatment in the workplace, such as bystander training and implicit bias training, have typically focused on mistreatment originating within the organization (ie, coworkers),^21^ vs mistreatment by patients and visitors. An employee-centered approach is less likely to influence harmful behavior by patients and visitors. Extant efforts to reduce mistreatment perpetrated by patients and visitors include published expectations of patient behavior and procedures for dismissing (ie, refusing to serve) abusive patients.^22^ However, it is unclear how effective these systems are in reducing the incidence of misbehavior. Addressing patients and visitors as sources of mistreatment will require a thoughtful approach that acknowledges multiple factors, including the power differential inherent in the physician-patient relationship, patients’ experiences of bias and mistreatment in the health care setting, and the primacy of patient experience metrics as a business imperative for health care organizations.

In our sample, there were disparities in the experience of mistreatment by gender, with women experiencing mistreatment at higher rates than men. Women were more likely to experience any form of mistreatment, as well as more likely to experience sexual harassment and verbal mistreatment. Previous studies have found higher rates of occupational distress among women physicians.^23,24,25,26^ These differences have been attributed to inequities in domestic responsibilities^25^ and to differences in the work environment.^27,28,29,30^ The increased rate of mistreatment experiences we found may be 1 modifiable factor in the work environment that contributes to the gender disparity in occupational well-being.

Prevalence of mistreatment also differed by race, with higher rates among the small number of physicians of color and those identifying as multiracial. Future research involving larger numbers of these racial groups are needed to assess replicability and significance of these observations. Nevertheless, these observations align with previous studies showing disparities in the experience of mistreatment by race and ethnicity among medical students and residents as well as numerous personal accounts of mistreatment that have been shared by physicians from underrepresented groups.^4,10,11,31^ More research is urgently needed.

Organizations have long sought to promote respect and to protect individuals from mistreatment through evidence-based interventions including implicit bias training, leadership development, anonymous reporting systems, and bystander training, among others. However, to our knowledge, there have been no studies in the health care field that measure how these protective mechanisms impact the occupational well-being of the people they are designed to protect. Our findings suggest that organizations may be able to influence the well-being of physicians by creating systems to ensure that they are treated with respect and dignity. Having these systems in place was significantly associated with reduced burnout, increased professional fulfillment, and reduced intent to leave the organization.

We explored bystander intervention as a specific example of protective environmental factors and found that it was independently associated with improved occupational well-being. This modest positive association between bystander intervention and occupational well-being was present both for physicians who reported mistreatment and those who did not. To our knowledge, this study is the first to demonstrate an association between perceived bystander intervention and occupational well-being.

Reducing mistreatment and enhancing protective systems has inherent ethical value, particularly considering that mistreatment is experienced inequitably based on race and gender. Initiatives that prioritize reducing mistreatment of women and physicians of color can help reduce gender- and race-based workplace inequities, and thereby support greater racial and gender diversity among physicians. In addition to these intrinsic values, our study suggests that such essential efforts may also result in benefits to patients, physicians, and health care organizations through reduction of burnout and its associated impacts. Physician burnout has been associated with harms to patients, including increased medical errors,^32,33^ poor patient experience of care,^34,35^ and worse patient outcomes in some studies,^36,37^ as well as harms to physicians, including increased rates of depression and substance abuse.^38,39^ Physician burnout threatens patient access to care through its association with increased rates of physician turnover and reduction in professional effort,^40,41^ which also impose additional recruitment costs on health care organizations.^14^ With occupational burnout rates of 40% to 60% documented in large, national studies over the last decade, physician burnout remains a major threat to physicians, patients, and health care organizations.^23^ Thus, any intervention that reduces the incidence of physician burnout is likely to yield dividends for the health care system across multiple dimensions.

### Strengths and Limitations

Our response rate of nearly 80% increases confidence that the sample is representative. There are also several limitations worth noting in this study. We used a binary gender classification owing to small sample size for other genders, which did not allow us to explore the experience of physicians who do not identify as male or female. The small number of non-White respondents precluded analysis of ethnicity, limiting the generalizability of our data on race. Caution is warranted in interpreting observed differences in descriptive data. No evaluation of statistical significance of observed difference between specific subgroup pairs was attempted due to the small number of underrepresented racial categories. Results indicate only that distribution of mistreatment was not equal across racial groups. The cross-sectional nature of our survey limited our ability to assess the directionality of the association between perceptions of protective systems and occupational well-being, and evaluation of the effectiveness of interventions was not possible. We did not assess the frequency or severity of mistreatment experiences. Further research is necessary to elucidate how frequency and severity of mistreatment impact outcomes. Given that the survey was promoted by the respondents’ employer (although administered by an independent surveyor), the potential exists for multiple types of response biases. Our study is also a single center experience which may affect the generalizability of some of our findings. Although it is unlikely the relationship between mistreatment and dimensions of occupational well-being are specific to this center, the prevalence of mistreatment may vary across centers and practice setting.

## Conclusions

This survey study found that workplace mistreatment was common for physicians. Patients and visitors were the most common source of mistreatment. We found disparities in mistreatment by gender and race, a strong negative association between mistreatment and occupational well-being, and a positive association between occupational well-being and protective workplace systems. These findings highlight the urgent need for organizations to put systems in place to reduce the incidence of mistreatment, and for more research to determine which systems will be most effective.

## Notes

Supplement. **eAppendix 1.** Methods for Developing the Mistreatment, Protection, and Respect (MPR) Measure **eAppendix 2.** MPR Measure Full Text **eTable 1.** Experience of Mistreatment, by Response Versus Missing Gender or Race Data **eTable 2.** Parameter Estimates from Regression Analyses of Associations of Mistreatment and Protective Factors with Standardized Scores for Burnout and Professional Fulfillment (PF)
