# Supplementary material for: Mistreatment Experiences, Protective Workplace Systems, and Occupational Distress in Physicians
Source: JAMA Netw Open. 2022 May 6;5(5):e2210768. doi: 10.1001/jamanetworkopen.2022.10768 (PMC9077480; doi:10.1001/jamanetworkopen.2022.10768)
Supplement: Supplement. — eAppendix 1. Methods for Developing the Mistreatment, Protection, and Respect (MPR) Measure eAppendix 2. MPR Measure Full Text eTable 1. Experience of Mistreatment, by Response Versus Missing Gender or Race Data eTable 2. Parameter Estimates from Regression Analyses of Associations of Mistreatment and Protective Factors with Standardized Scores for Burnout and Professional Fulfillment (PF) [file jamanetwopen-e2210768-s001.pdf]

Supplemental Online Content

Rowe SG, Stewart MT, Van Horne S, et al. Mistreatment experiences, protective workplace systems, and occupational distress in physicians. *JAMA Netw Open.* 2022;5(5):e2210768. doi:10.1001/jamanetworkopen.2022.10768

eAppendix 1. Methods for Developing the Mistreatment, Protection, and Respect (MPR) Measure

eAppendix 2. MPR Measure Full Text

eTable 1. Experience of Mistreatment, by Response Versus Missing Gender or Race Data

eTable 2. Parameter Estimates from Regression Analyses of Associations of Mistreatment and Protective Factors with Standardized Scores for Burnout and Professional Fulfillment (PF)

This supplemental material has been provided by the authors to give readers additional information about their work.

© 2022 Rowe SG et al. *JAMA Network Open.*

eAppendix 1: Methods for developing the Mistreatment, Protection, and Respect (MPR) measure

Four authors (SR, MBM, MPR, SVH) conducted literature searches for existing theoretical frameworks and relevant measures relating to mistreatment and protections from mistreatment in relation to well-being within health care. Focus groups were conducted with faculty exploring issues of gender and race in medicine. Two authors (SGR and MM) developed initial drafts of the 25-item grid assessing mistreatment and sources as well as the two questions about protective factors. Feedback on face and content validity, mistreatment categories, and mistreatment perpetrator categories was provided by: six other authors (MS, SVH, CP, MBM, ALP, MPR); one other physician with extensive experience assessing bias and mistreatment of physicians (EC); and a career ombudsman with 50+ years' experience working with organizations to prevent mistreatment and improve well-being (MPR). One author (MT) consulted on event frequency response options. After iterative feedback and improvement of the items, the scale was pilot tested in a diverse sample of 16 clinicians working in areas of diversity, equity and inclusion, to evaluate performance before administration to the study population.

© 2022 Rowe SG et al. *JAMA Network Open.*

eAppendix 2. Full Text of MPR Measure:

Have you experienced the following at work in the last 12 months and if so from whom? Please think about all interactions with patients, staff, colleagues, supervisors or others. Check all that apply.

A complaint or criticism related to your professionalism (appearance or behavior)
(select all that apply)

[ ]	Patient(s) (includes patient's family members)	[ ]	Colleague	[ ]	Nurse	[ ]	Other Staff	[ ]	Leadership (including supervisor)	[ ]	I did not experience this

A complaint or criticism related to the quality of your work (including safety or quality)
(select all that apply)

[ ]	Patient(s) (includes patient's family members)	[ ]	Colleague	[ ]	Nurse	[ ]	Other Staff	[ ]	Leadership (including supervisor)	[ ]	I did not experience this

Sexual harassment or abuse (including sexual comments or jokes, inappropriate sexual attention, unwelcome advances, requests for sexual favors, etc.)
(select all that apply)

[ ]	Patient(s) (includes patient's family members)	[ ]	Colleague	[ ]	Nurse	[ ]	Other Staff	[ ]	Leadership (including supervisor)	[ ]	I did not experience this

Verbal mistreatment or abuse (including use of slurs, insulting jokes or humor, name-calling, swearing, yelling, intimidation, verbal attacks, non-physical threats, warnings about retaliation)
(select all that apply)

© 2022 Rowe SG et al. *JAMA Network Open.*

[ ]	Patient(s) (includes patient's family members)	[ ]	Colleague	[ ]	Nurse	[ ]	Other Staff	[ ]	Leadership (including supervisor)	[ ]	I did not experience this

Physical intimidation, violence or abuse (including direct or implied threats of violence, threatening gestures or symbols, pushing, shoving, throwing things, assault including sexual or racial assault)

[ ]	Patient(s) (includes patient's family members)	[ ]	Colleague	[ ]	Nurse	[ ]	Other Staff	[ ]	Leadership (including supervisor)	[ ]	I did not experience this

To what extent is the following true at your organization?

	0	1	2	3	4
	**Not at all**	**To a small extent**	**To a moderate extent**	**To a great extent**	**To a very great extent**
**There are good systems in place to ensure that I am treated with respect and dignity**	[ ]	[ ]	[ ]	[ ]	[ ]
**Bystanders speak up or intervene if someone is mistreated**	[ ]	[ ]	[ ]	[ ]	[ ]

© 2022 Rowe SG et al. *JAMA Network Open.*

eTable1. Experience of mistreatment, by response versus missing gender or race data

		Gender			Race		
		**Gender available (n=1362)**	**Gender not available (n=143)**	**p-value**	**Race available (n=1213)**	**Race not available (n=292)**	**p-value**
**Sexual harassment or abuse,** n (%)	No	1252 (94.6)	57 (95.0)	>.99	186 (93.0)	1123 (94.8)	.37
	Yes	72 (5.4)	3 (5.0)		14 (7.0)	61 (5.2)	
**Verbal mistreatment or abuse,** n (%)	No	1038 (78.3)	50 (83.3)	.44	151 (75.1)	937 (79.1)	.24
	Yes	288 (21.7)	10 (16.7)		50 (24.9)	248 (20.9)	
**Physical intimidation, violence, or abuse,** n (%)	No	1265 (94.8)	57 (95.0)	>.99	194 (95.6)	1128 (94.7)	.74
	Yes	69 (5.2)	3 (5.0)		9 (4.4)	63 (5.3)	
**Any of above forms of mistreatment,** n (%)	No	1021 (76.4)	49 (81.7)	.43	920 (77.1)	150 (73.9)	.37
	Yes	316 (23.6)	11 (18.3)		274 (22.9)	53 (26.1)	

© 2022 Rowe SG et al. *JAMA Network Open.*

eTable 2: Parameter Estimates from Regression Analyses of Associations of Mistreatment and Protective Factors with standardized scores [a] for Burnout and Professional Fulfillment (PF)

	**Model 1a (Burnout)**<br>**Linear Regression**<br>**N = 1458**<br>**R^2 = 0.06**<br>**F (2, 1455) = 43.42**	**Model 2a (PF)**<br>**Linear Regression**<br>**N = 1479**<br>**R^2 = 0.04**<br>**F (2, 1476) = 29.18**
**Independent Variables**	**Beta (95% CI)**	**Beta (95% CI)**
Abuse (ref: No)		
Yes	0.57 (0.45, 0.70)	-0.47 (-0.59, -0.35)
Missing	0.18 (-0.04 0.40)	-0.06 (-0.27, 0.16)
	**Model 1bZ (Burnout)**<br>**Linear Regression**<br>**N = 1458**<br>**R^2 = 0.19**<br>**F (12, 1445) = 27.45**	**Model 2bZ (PF)**<br>**Linear Regression**<br>**N = 1479**<br>**R^2 = 0.22**<br>**F (12, 1466) = 34.77**
**Independent Variables**	**Beta (95% CI)[a]**	**Beta (95% CI)[a]**
Abuse (ref: No)		
Yes	0.27 (0.15, 0.39)	-0.10 (-0.21, 0.02)
Missing	0.12 (-0.17, 0.41)	0.14 (-0.14, 0.42)
Protective Systems (ref: To a very great extent)		
To a great extent	0.39 (0.22, 0.55)	-0.42 (-0.58, -0.26)
To a moderate extent	0.67 (0.48, 0.85)	-0.77 (-0.95, -0.59)

© 2022 Rowe SG et al. *JAMA Network Open.*

To a small extent	0.87 (0.63, 1.11)	-1.12 (-1.35, -0.89)
Not at all	1.23 (0.92, 1.54)	-1.35 (-1.65, -1.04)
Missing	0.36 (0.02, 0.70)	-0.47 (-0.80, -0.14)
Bystanders Speak Up (ref: To a very great extent)		
To a great extent	0.15 (-0.05, 0.35)	-0.16 (-0.36, 0.03)
To a moderate extent	0.16 (-0.05, 0.37)	-0.18 (-0.38, 0.03)
To a small extent	0.14 (-0.10, 0.38)	-0.23 (-0.46, 0)
Not at all	0.55 (0.26, 0.84)	-0.60 (-0.89, -0.31)
Missing	0.30 (-0.01, 0.61)	-0.44 (-0.74, -0.14)

[a] Dependent variable was transformed to Z scores so that beta coefficients represent standardized mean difference effect-sizes associated with independent variables

© 2022 Rowe SG et al. *JAMA Network Open.*
